# Prevalence of Prenatal Depression Symptoms Among 2 Generations of Pregnant Mothers: The Avon Longitudinal Study of Parents and Children

**DOI:** 10.1001/jamanetworkopen.2018.0725

**Published:** 2018-07-13

**Authors:** Rebecca M. Pearson, Rebecca E. Carnegie, Callum Cree, Claire Rollings, Louise Rena-Jones, Jonathan Evans, Alan Stein, Kate Tilling, Melanie Lewcock, Debbie A. Lawlor

**Affiliations:** 1 Centre for Academic Mental Health, Population Health Sciences, Bristol Medical School, University of Bristol, Bristol, United Kingdom; 2 Population Health Sciences, Bristol Medical School, University of Bristol, Bristol, United Kingdom; 3 Department of Psychiatry, University of Oxford, Oxford, United Kingdom; 4 Medical Research Council Integrative Epidemiology Unit, University of Bristol, Bristol, United Kingdom; 5 National Institute for Health Research Bristol Biomedical Research Centre, Bristol, United Kingdom

## Abstract

**Importance:**

Depression during pregnancy (prenatal depression) is common and has important consequences for mother and child. Evidence suggests an increasing prevalence of depression, especially in young women. It is unknown whether this is reflected in an increasing prevalence of prenatal depression.

**Objective:**

To compare the prevalence of depression during pregnancy in today’s young mothers with their mothers’ generation.

**Design, Setting, and Participants:**

In a longitudinal cohort study, we compared prenatal depressive symptoms in 2 generations of women who participated in the Avon Longitudinal Study of Parents and Children. Participants were the original mothers (recruited when they were pregnant) and their female offspring, or female partners of male offspring, who became pregnant. Both groups were limited to the same age range (19-24 years). The first generation of pregnancies occurred in 1990 to 1992 (n = 2390) and the second in 2012 to 2016 (n = 180). In both generations, women were born in the same geographical area (southwest England).

**Main Outcomes and Measures:**

Depressed mood measured prenatally using the Edinburgh Postnatal Depression Scale in self-reported surveys in both generations. A score of 13 or greater on a scale of 0 to 30 indicated depressed mood.

**Results:**

Of 2390 pregnant women in the first generation who were included in analysis (mean [SD] age, 22.1 [2.5] years), 408 (17%) had high depressive symptom scores (≥13). Of 180 pregnant women in the second generation who were included in the analysis (mean [SD] age, 22.8 [1.3] years), 45 (25%) had high depressive symptom scores. Having high depressive symptom scores was more common in the second generation of young pregnant women than in their mothers’ generation (relative risk, 1.51; 95% CI, 1.15-1.97), with imputation for missing confounding variable data and adjustment for age, parity, education, smoking, and body mass index not substantially changing this difference. Results were essentially the same when analyses were restricted to the 66 mother-offspring pairs. Maternal prenatal depression was associated with daughters’ prenatal depression (relative risk, 3.33; 95% CI, 1.65-6.67).

**Conclusions and Relevance:**

In this unique study of 2 generations of women who answered identical questionnaires in pregnancy, evidence was found that depressed mood may be higher in young pregnant women today than in their mothers’ generation. Because of the multiple and diverse consequences of prenatal depression, an increase in prevalence has important implications for families, health care professionals, and society.

## Key Points

**Question:**

Is the prevalence of depression in pregnancy increasing across 2 generations of the Avon Longitudinal Study of Parents and Children?

**Findings:**

In this 2-generation cohort study, evidence was found showing that depression in young pregnant women is higher today than in the 1990s.

**Meaning:**

The findings highlight the need for increased support for young pregnant women to minimize the potentially far-reaching effects of depression on mothers, their children, and future generations.

## Teaser

This cohort study uses data from the Avon Longitudinal Study of Parents and Children (ALSPAC) to compare rates of prenatal depression between women who were pregnant in 1990 to 1992 and a second generation of women who were pregnant in 2012 to 2016.

## Introduction

Depression has previously been estimated to affect approximately 10% to 15% of pregnant women,^1^ a similar magnitude to that found among postnatal women.^2^ Prenatal depression is associated with an increased risk of offspring emotional, behavioral, and cognitive difficulties.^3^ In addition, prenatal depression often continues after birth, where further risks to mothers’ health, parenting, and child development are observed.^3^ It has been estimated that the costs of perinatal mental illness in the United Kingdom alone are more than £8 billion (approximately $10.6 billion) each year, to which prenatal depression is a significant contributor.^4^ An increase in prenatal depression prevalence would represent a significant public health concern, with implications for current families and future generations alike.

There is evidence from routine data and population surveys that psychiatric service use and antidepressant prescriptions have increased in recent years.^5,6^ Whether these changes reflect less stigma and greater awareness of mental health, a genuine increase in overall population levels of depression, or both is unclear. Comparing population surveys using comparable tools to measure depression provides a more valid estimate of trends in depression prevalence; however, findings from such studies are inconsistent.^7,8,9,10,11^ One explanation for inconsistent findings is that the trends in prevalence of depression over time vary between different subgroups of the population, and studies vary in participant composition (ie, number of young women in the population studied). Indeed, evidence from population surveys (including the UK Office for National Statistics surveys^11^) suggests that depression in young women (aged 19-24 years) is increasing more than depression in other gender and age groups.^9,10,11^

We hypothesized that the increase of depression among young women will also be reflected during pregnancy. To our knowledge, this is the first study to compare prenatal depression prevalence in young women across time.

The Avon Longitudinal Study of Parents and Children (ALSPAC) is a prospective, multigenerational cohort that recruited pregnant women living in southwest England between 1990 and 1992 and has followed them, their partners, and their index children since then.^12,13^ In this analysis, ALSPAC-G0 participants are the original mothers (recruited when they were pregnant), and ALSPAC-G1 are female offspring participants, or female partners of male offspring participants, who became pregnant. The children of ALSPAC-G0 participants are now aged 23 to 25 years. In 2012 we began to recruit the next generation, ie, female ALSPAC-G1 offspring who became pregnant, female partners carrying offspring of male ALSPAC-G1, and any children of the original ALSPAC-G1 offspring. Depressive symptoms during pregnancy have been collected using identical methods in both ALSPAC-G0 participants and in the next generation of pregnancies in 2012 to 2016. This provides an opportunity to compare the levels of prenatal depression in contemporary young women with their mothers’ generation who were pregnant in the early 1990s.

We hypothesized that the frequency of prenatal depression would be higher in today’s sample of young pregnant mothers compared with rates in young women in the 1990s.

## Methods

### Participants

The ALSPAC-G0 group consisted of pregnant women whose delivery date fell between April 1, 1991, and December 31, 1992 (inclusive), and who resided in the county previously known as Avon in southwest England. Participants were recruited from 3 maternity units where every woman who attended prenatal clinics over a fixed period of time was invited, and most joined the study. The original participants totaled 14 541 pregnant women, of whom 13 988 had children alive at 1 year; these children are referred to as ALSPAC-G1. The G0 women, their partners, and their children (G1) have been followed since the early 1990s through questionnaires, detailed clinical assessments, and record linkage. Full details of the study have been previously reported,^12,13^ and further information can be found at http://www.bristol.ac.uk/alspac/. Ethical approval for the study was obtained from the ALSPAC Law and Ethics Committee and South West – Central Bristol National Health Service Research Ethics Committee, and participants gave written informed consent. We followed the Strengthening the Reporting of Observational Studies in Epidemiology (STROBE) reporting guideline in writing this article.

In 2012 we began recruiting and collecting data on the next generation, ALSPAC-G2, the children of the G1 participants and grandchildren of the originally recruited G0 women. At the time that we began the G2 study, we developed protocols for collecting data on any G1 participants who had become parents, were pregnant, or had a partner who was pregnant. As with the original study, we collected data on both parents (≥1 of whom was an ALSPAC-G1 participant) and their children.

The recruitment of G2 participants is through an open cohort. At the start of the study in 2012, some G1 participants had already become parents, and their children were entered into the study at any time (from early pregnancy on). The analysis for this specific article includes only G1 women recruited during pregnancy; of the total 442 G1 women who have become pregnant or G1 men who have a partner who is pregnant or who have become a parent, 180 provided depression data during pregnancy and were therefore eligible for this study. There were also 66 G0-G1 mother-offspring pairs in which both G0 and G1 women were pregnant at an age of 19 to 24 years and both had complete pregnancy depression data.

**Figure 1.**
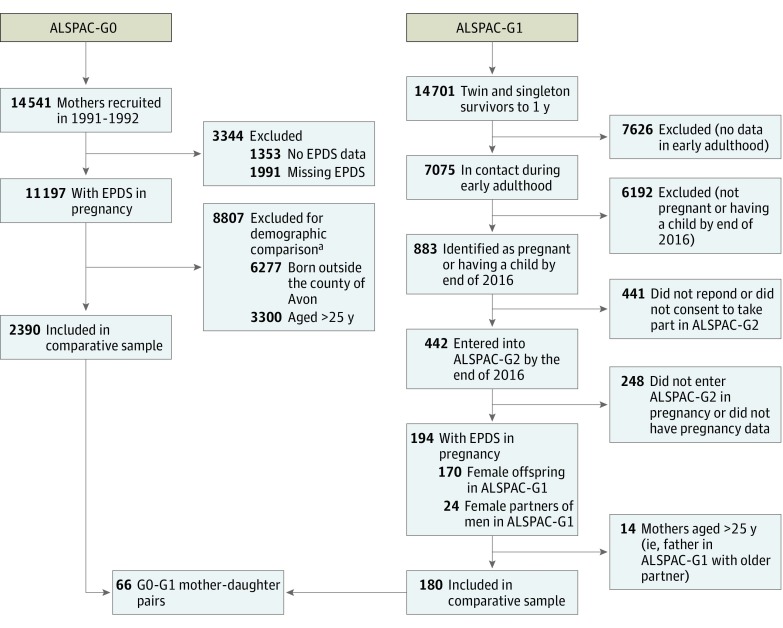
Flow Diagram Describing Numbers of Participants in Both Generations With Varying Data ALSPAC-G0 indicates original participants in the Avon Longitudinal Study of Parents and Children; ALSPAC-G1, offspring of ALSPAC-G0; ALSPAC-G2, offspring of ALSPAC-G1; and EPDS, Edinburgh Postnatal Depression Scale. ^a^Individuals could be excluded for more than 1 reason.

The focus of this study is on prenatal depression and therefore includes all G1 participants or their partners who were recruited during pregnancy between June 6, 2012, and December 31, 2016, and completed a depression measure at either of the pregnancy assessments (one at 18 weeks and the second at 32 weeks). Where women completed both assessments, we used the early pregnancy measure so that in all women we were using their first report of depression in pregnancy. We only included G0 mothers who were in the same age range as the G1 generation (ie, aged 19-24 years when they were originally recruited in pregnancy). The G1 participants, by definition of the ALSPAC initial recruitment, were born in Avon. Therefore, we also restricted the G0 sample to those born in Avon (approximately 50% of the original sample). Thus, our study uses data from the first pregnancy assessments of 2390 mothers from 1991 to 1992 (G0) and 180 mothers from 2012 to 2016 (G1), all of whom were aged 19 to 24 years at the time of pregnancy assessment, had the same measure of prenatal depression assessed at approximately the same time in pregnancy, and had been born in Avon (Figure 1).

### Measures

The Edinburgh Postnatal Depression Scale (EPDS)^14^ was used to measure levels of depression in both G0 and G1 mothers. The EPDS is a 10-item self-report measure designed to screen women for symptoms of depression both during and after pregnancy. High levels of depressive symptoms were defined as a score of 13 or greater on the EPDS, a threshold commonly used in practice and well validated with strong specificity and sensitivity against diagnostic tools.^14,15^ We also compared continuous symptom scores across generations.

We collected information on characteristics that are known to be associated with prenatal depression and that might differ between generations or have changed over the last 25 years to see whether differences between the 2 groups might be explained by any of these. With the exception of weight and height, similar protocols were used to collect these data in both generations. Age (in years), educational attainment (Advanced-Level qualifications [A-Levels], yes vs no), smoking (during pregnancy, yes vs no), and alcohol (any during pregnancy vs none) were obtained from questionnaires. Gestational age at the time of each assessment of depression was calculated from the gestational age at delivery and date of the assessment. In ALSPAC-G0 body mass index (BMI) (calculated as weight in kilograms divided by height in meters squared) was calculated from the woman’s retrospective report of her prepregnancy weight and measured height in early pregnancy. In ALSPAC-G1 both maternal weight and height were measured in early pregnancy by researchers.

### Statistical Analysis

Characteristics for the 2 groups are presented as percentages (categorical variables) or means (continuous variables). Differences in means or prevalence ratios with 95% confidence intervals were used to explore differences between generations. We used binary regression with a log-link function to estimate rate ratios comparing rates of depression in ALSPAC-G1 and ALSPAC-G0. We present unadjusted associations and associations adjusted for age, parity, education, smoking, alcohol, and BMI. Adjusted associations are presented separately for each characteristic to enable us to see the extent to which any of them explain any differences. We adjusted for gestational age when women completed the EPDS (early or late pregnancy).

We also investigated whether prenatal depression in G1 participants was associated with their own mothers’ depression during pregnancy.

In additional analyses we compared continuous mean scores and the unadjusted responses to each of the 10 items of the EPDS between the 2 groups of women to determine whether any differences in the overall score might be driven by specific symptoms of depression. We also repeated our main unadjusted analyses in the G0-G1 mother-offspring pairs only (n = 66). We also used multivariable multiple imputation to deal with missing covariable data in G1 as an additional sensitivity analysis (full methodological details are available in the eTable and eAppendix in the Supplement).

## Results

Of 2390 G0 women included in the analysis (mean [SD] age, 22.1 [2.5] years), 408 (17%) had high depressive symptom scores (≥13). Of 180 G1 women included in the analysis (mean [SD] age, 22.8 [1.3] years), 45 (25%) had high depressive symptom scores. The vast majority of G1 pregnancies were original female ALSPAC participants (163 of 180 [90.5%]), with the remaining 17 (9.5%) being pregnant female partners of a male ALSPAC-G1 participant. Within our total sample, there were 66 mother-offspring pairs.

Compared with their mothers’ generation, ALSPAC-G1 women were more likely to have achieved A-Levels and were less likely to smoke, but were more likely to take antidepressants (Table 1). Alcohol consumption during pregnancy was similar in the 2 generations. In unadjusted Poisson regression, the likelihood of having probable depression during pregnancy (EPDS score ≥13) was 1.51 times higher in ALSPAC-G1 mothers compared with ALSPAC-G0 (risk ratio [RR], 1.51; 95% CI, 1.15-1.97) (Table 2). In a model adjusting for age, BMI, smoking, parity, and education, the strength of the association increased to 1.77 (95% CI, 1.27-2.46). Following imputation for missing covariate data in G0 and using the same sample for both analyses (n = 2565) the adjusted RR was 1.90 (95% CI, 1.29-2.82), potentially highlighting some negative confounding. Restricting the analyses to the 66 mother-offspring pairs and accounting for clustering of pairs, we found a virtually identical association, but with wider a confidence interval (RR, 1.46; 95% CI, 0.88-2.42).

**Table 1.** Comparison of Maternal Pregnancy Characteristics and Prenatal Depression in 2 Generations of Women

Characteristic	No. With Outcome/Denominator^a^ (%)		Mean Difference or Risk Ratio (95% CI)
	G0: Pregnant 1991-1992 (n = 2390)	G1: Pregnant 2012-2016 (n = 180)	
Age, mean (SD), y	22.1 (2.5)	22.8 (1.3)	0.67 (0.30-1.04)
Nulliparous	2311/2558 (90)	108/176 (61)	4.08 (3.28-5.10)
A-Level or higher education attainment	328/2268 (15)	52/146 (36)	2.50 (2.01-3.11)
Smoking in pregnancy	802/2224 (36)	27/147 (18)	0.51 (0.36-0.72)
Antidepressants in pregnancy	43/2699 (2)	23/180 (13)	8.02 (4.95-13.00)
Prenatal depression	408/ 2390 (17)	45/180 (25)	1.51 (1.15-1.97)

Abbreviations: ALSPAC-G0, original participants in the Avon Longitudinal Study of Parents and Children; ALSPAC-G1, offspring of ALSPAC-G0.

^a^ Denominators vary because of missing data.

**Table 2.** Differences in Rates of Prenatal Depression Between 2 Generations of Women

Adjusted for	Early Pregnancy Depression		
	No.	Unadjusted Risk Ratio (95% CI)	Adjusted Risk Ratio (95% CI)
Age	2565	1.51 (1.15-1.97)	1.70 (1.30-2.22)
Nulliparity	2513	1.69 (1.24-2.23)	1.65 (1.24-2.18)
Education	2229	1.70 (1.30-2.23)	1.70 (1.30-2.24)
Smoking	2183	1.60 (1.18-2.17)	1.70 (1.26-2.41)
Body mass index^a^	2315	1.88 (1.40-2.53)	1.76 (1.29-2.40)
All confounding variables above	1714	1.78 (1.25-2.55)	1.77 (1.27-2.46)
After imputation for missing covariate data	2565	1.51 (1.15-1.97)	1.90 (1.29-2.82)

^a^ Calculated as weight in kilograms divided by height in meters squared.

Maternal (G0) prenatal depression was associated with daughter’s (G1) prenatal depression. Of G1 participants whose mothers were depressed in pregnancy, 54% were depressed themselves, compared with only 16% of G1 participants whose mothers were not depressed prenatally (RR, 3.33; 95% CI, 1.65-6.67).

**Figure 2.**
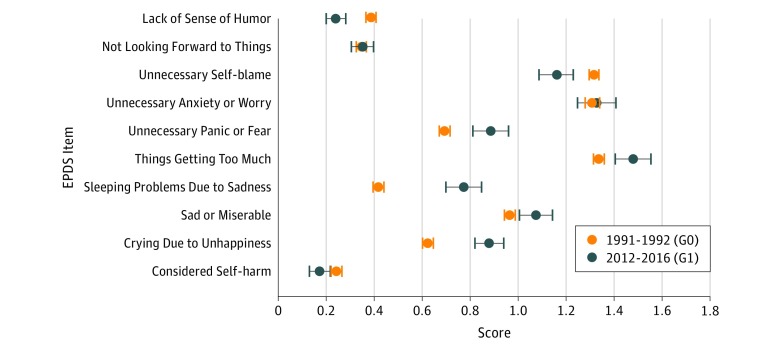
Mean Scores for Individual Items on the Edinburgh Postnatal Depression Scale (EPDS) for 2 Generations of Women During Pregnancy G0 indicates the first generation studied in the Avon Longitudinal Study of Parents and Children (ALSPAC); G1, second generation studied in ALSPAC; and error bars, 95% CI. The individual EPDS item scores all increased in G1 mothers, with the exception of unnecessary self-blame and lack of sense of humor, which were more common in G0 participants, and considering self-harm, which was similar in both groups.

Comparing mean scores using linear regression found that overall scores in G1 were on average 0.96 (95% CI, 0.19-1.74; *P* = .02) higher than scores in G0. The individual EPDS item scores all increased in G1 mothers, with the exception of unnecessary self-blame and lack of sense of humor, which were more common in ALSPAC-G0 participants, and considering self-harm, which was similar in both groups (Figure 2). Notably higher levels in ALSPAC-G1 participants were seen for items relating to feeling overwhelmed and explicit symptoms of crying and difficulty sleeping.

## Discussion

The results suggest that prenatal depression is on average 51% more common among young mothers in the current generation of the ALSPAC cohort than during their mothers’ generation 25 years ago. This finding persists after adjusting for factors that differ across generations and in analyses restricted to mother-offspring pairs. Given the costs associated with prenatal depression and consequences for the mother, the child, and the wider society, an increase in prevalence is important to service provision and public health regardless of whether the increase is specific to pregnancy. The findings highlight the need for further research to elucidate the reasons behind this intergenerational trend and reduce negative effects.

### Strengths

One of the main strengths of our study comes from the use of 2 generations within the same cohort, with both generations living in the same residential area. The measures of depression were identical, and timing of measures were similar. Characteristics that differed between the generations that might explain differences in depression were also largely measured similarly between the 2 generations, and adjusting for these did not materially alter the difference across generations.

### Limitations

The sample size for the ALSPAC-G1 group is small, as relatively few of the ALSPAC-G1 participants had become pregnant at the time data were collected, especially given that age at first birth increased from 28 years in 1992 to 30 years in 2012 in the United Kingdom.

Given the current interest in increasing rates of depression in young women and the importance of prenatal depression, we feel these results are important. Nonetheless, further replication of these findings would be valuable.

We were specifically interested in young mothers between 19 and 24 years of age because this has been identified as a high-risk group, but we cannot assume that our findings are generalizable to older pregnant women. The ALSPAC population largely consists of white European individuals, and we cannot assume similar findings in other ethnic groups.

People with mental health problems are less likely to take part in research. However, we think it unlikely that a 51% increase in the rate of depressed mood can be explained by contemporary pregnant women who are depressed responding more frequently than pregnant women who were similarly depressed in the early 1990s. To be included in this study, G1 women had to be recruited during their pregnancy. This means that for many of the eligible G1 participants we did not have pregnancy data. We have matched and controlled for maternal age; however, there may still be some differences based on different selection strategies for participants. Because of these differences, we do not have pregnancy data on those G1 participants who became pregnant before 2012 (at the younger age of 18-20 years). Given that very young age at pregnancy is a risk factor for depression, this would suggest that, if anything, depression in G1 is underestimated and the increase could be greater.

There are additional differences between generations that we could not account for, which could explain the difference we have observed. For example, although both G0 and G1 women were in the same age range (19-24 years), the average age of motherhood today is higher than in the 1990s.^16^ Comparatively, therefore, the ALSPAC-G1 participants may be having children at a younger age than their peers relative to their mothers’ generation, which may result in more social isolation or stigma and could be a potential source of unmeasured explanation for the difference. However, this would be a potential underlying mechanism for the increase in prenatal depression in young women rather than a source of confounding.

Using this unique data source, we also found that maternal (G0) depression during pregnancy was a strong risk factor for prenatal depression in the G1 women (RR, 3.33). This risk of intergenerational prenatal depression exceeds the unadjusted association for intergenerational depression at other time points in this sample (odds ratio, 1.5 at age 18 years) based on previous publications.^3^ However, to confirm any specificity, future studies would need to compare mother-daughter associations between depression at different timings and the extent to which the daughters’ prenatal depression was related to a continuation of depression before pregnancy as opposed to a prenatal onset. Given that depression at any timing is highly correlated, larger samples than we currently have available are needed to have sufficient statistical power to disentangle specific timing effects.^3^ If, however, a prenatal-specific association is confirmed in other samples, it may highlight a specific intergenerational risk of depression during pregnancy, with potential genetic, biological (eg, hormonal or placental function), or environmental mechanisms that require further investigation.^17^

### Mechanisms

The findings in this study mirror the more general increase in depression among young women that has been recently reported. If the increase in the prevalence of prenatal depression over the past 25 years is confirmed, it is important to understand the potential changes in society and lifestyle that may have contributed to the observed increase. Chronic stress, sleep deprivation, eating habits, sedentary lifestyle, and the fast pace of modern life may be contributing to an increasing prevalence of depression among young people generally.^18^ The impact of such changes may be amplified when a woman becomes pregnant. This generation of young women has also experienced rapid change in technology, internet, and social media use, which has been associated with increased feelings of depression and social isolation and changes to social relationships.^19,20^

Beyond the background mechanisms for increasing depression prevalence among young people, pregnant women are likely to face additional pressures. First, as compared with the 1990s, the proportion of mothers working has increased substantially,^21^ and inflexible work arrangements and work pressure are associated with greater depressive symptoms in mothers.^22^ Difficulties balancing work and home may be increasing, and this may be reflected by the increase of G1 women reporting “things are getting too much” compared with their mother’s generation (G0) in our sample (Figure 2). Financial pressures, which evidence suggests are also associated with depression during pregnancy,^23^ are also arguably greater today, adding further pressure to working mothers.

## Conclusions

Using a unique data source, we present evidence that depression in young pregnant women is higher today than in the 1990s. There are a number of plausible explanations for this phenomenon that deserve further investigation in future research to guide prevention and treatment. The findings highlight the need for increased screening and resources to support young pregnant women and minimize the potentially far-reaching impact of depression on mothers, their children, and future generations.

## Notes

Supplement. **eTable.** Numbers of Missing for Each Covariate **eAppendix.** Further Information Regarding Missing Data **eReference**
