# Supplementary material for: Prevalence of Prenatal Depression Symptoms Among 2 Generations of Pregnant Mothers: The Avon Longitudinal Study of Parents and Children
Source: JAMA Netw Open. 2018 Jul 13;1(3):e180725. doi: 10.1001/jamanetworkopen.2018.0725 (PMC6324308; doi:10.1001/jamanetworkopen.2018.0725)
Supplement: Supplement. — eTable. Numbers of Missing for Each Covariate eAppendix. Further Information Regarding Missing Data eReference [file jamanetwopen-1-e180725-s001.pdf]

Supplementary Online Content

Pearson RM, Carnegie RE, Cree C, et al. Prevalence of prenatal depression symptoms among 2 generations of pregnant mothers: the Avon Longitudinal Study of Parents and Children. *JAMA Netw Open*. 2018;1(3):e180725. doi:10.1001/jamanetworkopen.2018.0725

eTable. Numbers of Missing for Each Covariate

eAppendix. Further Information Regarding Missing Data

eReference

This supplementary material has been provided by the authors to give readers additional information about their work.

© 2018 Pearson RM et al. *JAMA Network Open.*

eTable. Numbers of Missing for Each Covariate

	Missing Data
Age	0
Nulliparity	52
Education	336
Smoking	382
Alcohol	64
BMI	250

eAppendix. Further Information Regarding Missing Data

We imputed for missing data because a complete case analytical approach can lead to biased results if the data are not missing completely at random (White et al 2011). For example, in this study the missing data is likely to follow a systematic pattern relevant to our hypothesis. Those mothers who are depressed are more likely to fail to complete surveys. However, we can correct for this bias by using available data to predict the values of those with missing data in ALSPAC –G0 covariate data, and we are confident in our ability to build an adequate imputation model for these missing data due to the wealth of auxiliary measures that can be employed for this purpose. For example, given that there is substantial information on socio-demographic variables in ALSPAC that predict missingness, missing information can be assumed dependent on observed data (missing at random assumption). We employed a fully conditional specification as implemented in the MI chained algorithm in STATA 13 using all variables described in the analyses and additional socio-demographic indicators of missingness (list available on request) to predict missing data across 100 imputed datasets. Monte Carlo errors were less than 10% of the standard error and FMI values were no larger than 0.8 (White 2011). The imputation method is based on regression equations to predict the missing variable. Therefore, the unique associations between *each* imputed variable and the predictor variables are used and every imputed variable is imputed using a unique set of regression equations. We imputed up to a sample with at least one measure of maternal depression in pregnancy of n= 2565, the extent of missing by individual co-variates is described in the eTable.

© 2018 Pearson RM et al. *JAMA Network Open.*

eReference

1. **White P, Royston IR**. (2011) Multiple imputation by chained equations (MICE): Implementation in Stata. Journal of Statistical Software. Vol 45, Issue 4

© 2018 Pearson RM et al. *JAMA Network Open.*
